# The COMFORTage project: study protocol for the integration of multiple sources towards personalised preventions at Ace Alzheimer Center Barcelona

**DOI:** 10.3389/fdgth.2025.1633507

**Published:** 2026-01-26

**Authors:** Sergi Valero, Andrea Miguel, Josep Blazquez-Folch, Berta Calm, Montserrat Alegret, Ariadna Solivar, George Manias, Athos Antoniades, Nelina Angelova, Despina Psimaris, Sofia Segkouli, Amèrica Morera, Natalia Tantinya, Maitee Rosende-Roca, Amanda Cano, Maria Victoria Fernández, Pilar Sanz-Cartagena, Miren Jone Gurruchaga, Lluís Tárraga, Mercè Boada, Agustín Ruiz, Marta Marquié, and COMFORTage Consortium

**Affiliations:** 1 Ace Alzheimer Center Barcelona, Universitat Internacional de Catalunya, Barcelona, Spain; 2 Networking Research Center on Neurodegenerative Diseases (CIBERNED), Instituto de Salud Carlos III, Madrid, Spain; 3 Department of Digital Systems, University of Piraeus, Piraeus, Greece; 4 Stremble Ventures LTD, Limassol, Cyprus; 5 Innovation Sprint srl, Clos Chapelle-aux-Champs, Brussels, Belgium; 6 Maggioli Spa, Athens, Italy; 7 Centre for Research and Technology Hellas, Information Technologies Institute (CERTH/ITI), Thessaloniki, Greece; 8 Glenn Biggs Institute for Alzheimer’s & Neurodegenerative Diseases, University of Texas Health Science Center, San Antonio, TX, United States; 9 Department of Microbiology, Immunology and Molecular Genetics, Long School of Medicine, University of Texas Health Science Center, San Antonio, TX, United States

**Keywords:** Alzheimer's disease, artificial intelligence, digital health, early diagnosis, mild cognitive impairment

## Abstract

**Introduction:**

Ageing is accompanied by gradual biological and cognitive changes that increase vulnerability to chronic diseases and neurodegenerative conditions. As populations age, dementia prevalence continues to rise, highlighting the need for earlier detection and personalised prevention strategies. Against this background, the COMFORTage project, funded by Horizon Europe, brings together a multidisciplinary consortium across 12 countries to advance innovative, scalable solutions for dementia care. By integrating digital platforms, biomarker research, and precision medicine, COMFORTage seeks to develop artificial intelligence (AI)–driven tools that support more precise and adaptive interventions. Central to this effort are the Virtualized AI-Based Healthcare Platform and Patient Digital Twins, which enable personalised monitoring and decision support. Within this framework, Pilot 3 at Ace Alzheimer Center Barcelona focuses on individuals with mild cognitive impairment and mild Alzheimer's disease dementia, evaluating the effects of cognitive and functional stimulation and contributing multimodal data to optimise the AI platform.

**Methods:**

Pilot 3 is a randomised, open-label study involving retrospective and prospective datasets. Participants undergo clinical, genetic, neuropsychological, cerebrospinal fluid (CSF) and plasma biomarker assessments, magnetic resonance imaging (MRI), and spontaneous speech analysis. The primary outcomes assess cognitive decline using composite scores from the Neuropsychological Battery used in Ace (NBACE), targeting attention, memory, visuospatial/perceptual functions, executive functions, and language, over a two-year follow-up. Three digital platforms provided by the consortium will be used as cognitive and functional stimulation tools for participants. The intervention's effects on cognitive decline will be evaluated through changes in NBACE composite scores. Secondary objectives include assessing impacts on physical, psychological, social, and functional well-being; examining associations between biological variables and cognitive changes; and analyzing spontaneous speech as a remote, scalable proxy for cognitive status.

**Discussion:**

Findings from Pilot 3 will contribute to COMFORTage's broader mission, offering critical insights into the scalability and real-world implementation of AI-powered dementia care solutions. This integrated approach highlights the potential of precision medicine and advanced digital tools to elevate global standards in dementia management.

**Clinical Trial Registration:**

identifier NCT07031167.

## 1 Introduction

Ageing is inherently associated with gradual changes in brain function, metabolism, and physical performance. While some older adults adapt well and maintain cognitive and physical well-being, others experience a decline that increases their vulnerability to health complications. Regardless of baseline health status, these age-related changes can predispose individuals to both cognitive and functional deterioration. Early detection of risk factors is crucial for developing proactive interventions that promote healthy ageing (1). A rapidly ageing population amplifies these challenges, as increasing longevity is accompanied by higher rates of chronic disease, frailty, and cognitive impairment, leading to substantial social and economic consequences. Preserving cognitive reserve and functional independence has therefore become a key priority, underscoring the need for earlier, personalised strategies to detect disease processes before the onset of clinical dementia (2).

As these age-related changes progress, some individuals develop neurodegenerative conditions, most notably Alzheimer's disease (AD), which markedly accelerates cognitive and functional decline. When such disorders coexist with frailty, their combined impact further increases the risk of disability, loss of autonomy, and reduced quality of life (3, 4).

AD is the leading cause of dementia, accounting for 60%–80% of cases worldwide, and its prevalence continues to rise as improvements in healthcare and living standards have led to ageing populations (5). The number of people living with dementia worldwide in 2019 was estimated at 57 million and is projected to increase to 153 million by 2050 (6). In Europe, approximately 9.8 million people were living with dementia in 2018, and this number is projected to nearly double to around 18.8 million by 2050 (7). AD has become not only a growing medical challenge but also a major socio-economic concern (7, 8). Addressing this burden requires integrated and scalable strategies that enable earlier detection and more personalised prevention of dementia.

The disease follows a continuum that begins with asymptomatic pathological changes, progresses through mild cognitive impairment (MCI), and culminates in severe dementia. MCI, which manifests as measurable cognitive decline that does not yet significantly disrupt daily life, is widely recognised as a prodromal stage of AD (9, 10). Given the elevated risk of progression to dementia in certain individuals with MCI, early detection and targeted interventions are critical (11–13). This underscores the importance of developing multimodal approaches for early and precise identification of neurodegenerative changes.

Addressing these growing challenges requires pioneering approaches that transcend traditional healthcare models. The COMFORTage project, funded by the European Union under the Horizon Europe Programme, is an innovative European initiative running from 2024 to 2027 that seeks to address the complex interplay between ageing, dementia, and frailty through the integration of clinical expertise, technological innovation, and community engagement. Bringing together 39 partners across 12 countries, COMFORTage unites expertise from diverse disciplines to foster a truly collaborative and multidisciplinary approach. This multidisciplinary collaboration aims to establish a pan-European framework for community-based, integrated, and person-centered solutions for age-related health challenges (14).

The project supports older adults experiencing cognitive and functional decline due to neurodegenerative disorders, frailty, or other age-related impairments. These conditions are major contributors to disability and dependence, imposing a growing burden on individuals, caregivers, and healthcare systems. Within this framework, COMFORTage applies artificial intelligence (AI) and digital innovation to improve prevention, early detection, and personalised care, while promoting digital inclusion and collaboration among healthcare providers, patients, and caregivers. The project underscores the need for adaptable, multifactorial strategies that address the diverse and evolving needs of ageing populations through inclusive and person-centred interventions.

Several large multicentre initiatives have advanced different aspects of the early-detection-to-care pathway in AD, including the development of AI-based risk models, the validation of digital measures using smartphones and wearables, the creation of well-characterised longitudinal cohorts, and the implementation of lifestyle-based prevention or home-monitoring approaches (15–18). While these efforts have each contributed valuable knowledge, they generally focus on single components of the dementia continuum. In contrast, COMFORTage brings these complementary approaches together within a single, coherent framework that supports early detection, personalised intervention, and coordinated care, and evaluates their real-world feasibility across 13 community-based pilots.

Among the project's key innovations is the Virtualized AI-Based Healthcare Platform (VHP), a cornerstone of COMFORTage designed to improve the management of dementia, frailty, and other age-related conditions through secure, data-driven, and personalised care solutions. The platform centralizes artificial intelligence resources that support risk-factor analysis, early diagnosis, and individualised clinical decision-making across mental and physical domains of ageing.

The VHP integrates data from multiple sources—such as electronic health records, wearable devices, and real-world evidence—into unified Holistic Health Records (HHRs), creating comprehensive and interoperable patient profiles. The HHR concept was originally introduced by the iHELP project for holistic detection, early risk prediction, and monitoring of pancreatic cancer (19). Built on a secure, General Data Protection Regulation–compliant architecture, the VHP ensures data privacy, transparency, and traceability. At its core is the Integrated Care Model Library (ICML), a repository of AI-driven tools and resources designed to address the complexities of age-related diseases. By leveraging genomic, behavioural, and clinical data, the ICML enables precision in prevention, intervention, and treatment, providing clinicians with predictive and explainable tools to support decision-making. The platform also includes Patient Digital Twins, which generate individualised digital models that allow continuous monitoring and personalised adaptation of care plans.

Complementary assistive and educational modules are also integrated into the VHP to strengthen digital engagement and support self-management. These include the Training and Educational Toolkit (TET), which provides accessible learning materials and interactive resources—such as cognitive training games and practical guidance on using digital health tools—to help users build skills relevant to their care. These resources are delivered through the Training and Educational Marketplace (TEM), a secure environment that hosts a curated selection of applications and uses a recommendation system to suggest tailored content based on individual needs and preferences. Together, the TET and TEM enhance digital literacy among older adults, caregivers, and healthcare professionals, reinforcing the VHP's role as an integrated platform for prevention, monitoring, and personalised intervention.

The COMFORTage project employs a framework of 13 pilot studies distributed across five research clusters to evaluate and refine the VHP and ensure its applicability across diverse populations. These pilots serve as test beds for new technologies, methodologies, and interventions, focusing on scalability and adaptability to regional healthcare systems and cultural contexts. The pilots are strategically located across Europe and involve key academic and clinical partners.

The five clusters integrating the different pilots are: Cluster A) focuses on identifying causal risk factors and developing community-based strategies for early diagnosis and prevention; Cluster B) examines the relationship between dementia and comorbid conditions, broadening the understanding of factors that influence progression; Cluster C) develops personalised interventions to delay progression and improve quality of life; Cluster D) addresses physical health and safety, focusing on neuromechanics and fall prevention; and Cluster E) explores the role of digital innovation hubs and living labs in promoting active ageing and social inclusion.

Ace Alzheimer Center Barcelona (Ace) leads Pilot 3 within Cluster A of the COMFORTage project, which focuses on early detection and cognitive stimulation for individuals with MCI and mild AD dementia. The pilot plays a pivotal role in both the clinical validation of the consortium's interventions and the development of the VHP through the contribution of retrospective and prospective datasets.

In this study, participants are randomly assigned to active or control groups and followed longitudinally for two years to monitor cognitive and functional decline. Assessments include clinical, neuropsychological, genetic, cerebrospinal fluid (CSF), plasma biomarker, and magnetic resonance imaging (MRI) data, enabling an integrated analysis of biological and cognitive interactions (12, 20). These multimodal biomarkers offer complementary information across the disease continuum, supporting more accurate characterization of both early pathology and ongoing neurodegeneration (21, 22). Periodic speech-based digital assessments further complement these measures by providing remote and ecologically valid indicators of subtle cognitive and behavioural change (23–25).

By combining traditional and digital measures, the Ace Pilot aims to evaluate the efficacy of cognitive and functional stimulation interventions while simultaneously generating high-quality, multimodal data to train and validate the AI models underpinning the VHP. Primary outcomes focus on cognitive decline, while secondary measures assess quality of life (QoL), physical activity, and satisfaction with the intervention.

## 2 Methods

### 2.1 Study design

The Ace Pilot (Pilot 3) is a randomised, open-label, two-year clinical trial comparing outcomes between an active group receiving a structured cognitive and functional stimulation program and a control group receiving standard care. In parallel, retrospective datasets from Ace's existing cohorts, including clinical, neuropsychological, genetic, CSF and plasma biomarkers, and MRI data, will be shared with the consortium to support the development and validation of the VHP's AI algorithms.

### 2.2 Objectives

The Ace Pilot aims to enhance the quality of life and overall well-being of individuals with MCI and mild AD dementia by prospectively evaluating a structured cognitive and functional stimulation program, while simultaneously generating high-quality retrospective datasets to support the development and validation of the VHP's AI-based models.

#### 2.2.1 Primary clinical outcome

To evaluate cognitive decline as the primary efficacy outcome between the active and control groups after a two-year follow-up. Cognitive change will be quantified using composite scores derived from the Neuropsychological Battery used in Ace (NBACE) (26, 27), covering five cognitive domains: attention, memory, visuospatial/visuoperceptual functions, executive functions, and language (24).

#### 2.2.2 Translational objectives

Patient well-being and intervention impact: To examine the intervention's effects on participants’ physical, psychological, social, and functional well-being, providing a holistic understanding of health, lifestyle, and care needs.
Biological and cognitive interactions: To investigate the relationship between biological markers, including genetic profiles, CSF and plasma biomarkers, and MRI measures, and cognitive decline, and to identify the conditions under which e-health interventions are most effective. Plasma biomarkers will be measured at baseline, year 1, and year 2 to track longitudinal changes.
Spontaneous speech analysis: To employ automated speech processing for analysing spontaneous speech as a remote and scalable proxy for cognitive status, complementing traditional neuropsychological assessments (23, 24).
AI platform development: To contribute to the generation of multidimensional datasets that integrate clinical, biological, cognitive, and patient-reported outcomes in order to refine and validate the VHP's AI models, and to characterize the intervention's efficacy across diverse metrics for the benefit of participants and caregivers.

### 2.3 Study participants

**Figure 1:**
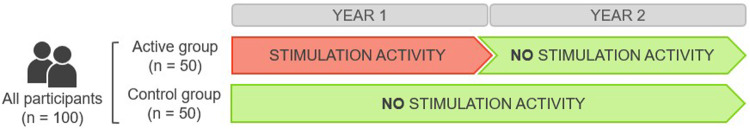
Study design and participant flow for pilot 3 at Ace.

The study participants for the Ace Pilot will consist of 100 individuals aged 60–85, divided into two groups of 50 participants each (active and control groups), see Figure 1. Eligible participants will be diagnosed with MCI according to Petersen criteria (13, 28) with a Clinical Dementia Rating (CDR) score of 0.5 (29) or mild AD dementia as defined by the National Institute on Aging-Alzheimer's Association (NIA-AA) criteria (30) with a CDR score of 1 (29).

Participants will follow a standardised protocol, with a consensus diagnosis determined by the research team based on a comprehensive evaluation, including neurological, neuropsychological, and social assessments (31). Additionally, participants must show proficiency in using digital devices such as mobile apps, tablets, or computers, as these tools are essential for both the intervention and monitoring processes.

The sample size was defined based on feasibility considerations, reflecting the expected number of eligible individuals who can be recruited during the study period. This estimate considers the typical annual flow of newly assessed patients who undergo the full diagnostic protocol, including neuropsychological evaluation, CSF and MRI, and who are able to attend regular on-site sessions at the centre. As a result, the target of 100 participants represents a realistic and achievable recruitment goal within the operational context of the Memory Unit.

In accordance with Spain's Data Protection Law (Organic Law 3/2018), all participants were informed by a neurologist about the study's goals and procedures before signing an informed consent form. Patient privacy and data confidentiality were safeguarded in compliance with applicable regulations.

Exclusion criteria include a history of medical conditions that could confound cognitive assessments or hinder participation, such as traumatic brain injury, severe depression, stroke, or brain tumors. Participants with significant uncorrected visual or auditory abnormalities, those without access to a digital device or internet connection, and individuals unable to attend regular in-person sessions at Ace will also be excluded.

### 2.4 Scheduled visits and assessments

Participants will be prospectively recruited at Ace's Memory Unit. The majority of patients in this unit are beneficiaries of the Catalan public health system and are typically referred by their general practitioners. Both the active and control groups will follow identical evaluation procedures, as outlined in Table 1, ensuring consistency and comparability in data collection and assessment across the study population.

**Table 1:** Overview of scheduled visits and assessments in pilot 3.

Evaluation/sample		Pre-intervention	Follow-up year 1	Follow-up year 2
Neurology		✓	✓	✓
Neuropsychology		✓	✓	✓
Nursing		✓	✓	✓
Spontaneous speech		✓^a^	✓^a^	✓^a^
MRI		✓	✕	✕
Fluid biomarkers	Plasma	✓	✓	✓
	CSF	✓	✕	✕
	Saliva	✓	✕	✕
Genotyping (PRS and *APOE* ɛ4)		✓	✕	✕
Cognitive and functional stimulation activity		✕	✓^b^	✕
Self-reported data		✓	✓	✓

MRI, magnetic resonance imaging; CSF, cerebrospinal fluid; PRS: Polygenic Risk Score.

^a^ Every 3–4 months remotely.

^b^ Weekly during the first year of follow-up, done remotely and in person.

Clinical, neuropsychological, and biological data will be systematically collected throughout the study to provide a comprehensive evaluation of participants’ progress and the intervention's impact. Clinical and neuropsychological assessments will be conducted in a face-to-face setting at the three critical time points: baseline (pre-intervention) and one-year and two-year follow-ups. These assessments will yield essential insights into participants’ cognitive and functional status over time, enabling a detailed analysis of the intervention's efficacy. Additionally, self-reported data will be gathered via standardised questionnaires during participants’ regular visits as part of the intervention activities. These face-to-face sessions will be carried out during the first year of the project, allowing consistent monitoring of clinical conditions, neuropsychological performances, and subjective experiences, such as quality of life and activity levels. The second-year follow-up is designed to extend the monitoring period and assess the sustainability of the intervention's effects after its conclusion, providing relevant insights into medium-term outcomes. At this final stage, the same clinical, neuropsychological, and self-reported measures are reassessed.

Biological data will also play a central role in this pilot study. Baseline collections will include genetic, CSF, and MRI data to establish a comprehensive understanding of participants’ biological profiles. Furthermore, plasma samples will be obtained not only at baseline but in the first and second year of follow-up, providing valuable longitudinal data to investigate correlations between dynamic variations in plasma biomarkers and cognitive outcomes.

To complement these assessments, spontaneous speech data will be remotely recorded every 3–4 months. This periodic speech sampling offers a scalable and non-invasive approach to monitoring cognitive changes over time and provides an additional source of information alongside face-to-face assessments and self-reported outcomes.

#### 2.4.1 Neurological evaluation

The assessment consists of a structured anamnesis conducted with both the patient and a caregiver (informant). A comprehensive neurological examination is performed, including a detailed medical history alongside the administration of several standardised tests to assess cognitive, functional, and neuropsychiatric status. These include the Mini-Mental State Examination (MMSE) (32), the Memory test of the Spanish version of the 7 Min Screen Test (33), the Hachinski Ischemia Scale (34), the Spanish version of the Neuropsychiatric Inventory Questionnaire (35), the Global Deterioration Scale (GDS) (36), the CDR (29), and the Blessed Scale (37).

Additionally, specific comorbidities are systematically assessed, including neurological, psychiatric, rheumatologic, endocrine-metabolic, cardiovascular, respiratory, nephrological, and autoimmune conditions. Other relevant factors, such as history of alcoholism, prior oncologic diagnoses, and traumatic brain injury, are also recorded. A detailed medication registry is systematically maintained, documenting all medications taken by participants. The assessment includes the Cardiovascular Risk Factors, Aging, and Incidence of Dementia (CAIDE) score (38) and the Cumulative Illness Rating Scale (CRIS) (39). These variables are not only critical for characterizing the patient population, but also for inclusion in exploratory analyses to examine potential interactions and confounding effects on the study outcomes.

#### 2.4.2 Neuropsychological assessment

The NBACE is a comprehensive tool designed to assess multiple cognitive, emotional, and functional domains. Table 2 provides an overview of all domains and their corresponding tests, offering a clear and concise summary of the full assessment structure. This table serves as the primary reference for the components included in the NBACE.

**Table 2:** Neuropsychological battery used in Ace (NBACE), assessment structure across cognitive and functional domains.

Category	Measurement tool/description
Reality orientation	Temporal, spatial and personal orientation tests
Attention & working memory	Digital Forward subtest of the Wechsler Adult Intelligence Scale, Third Edition (WAIS-III) (73)
Verbal learning & memory	Word List subtest from the Wechsler Memory Scale, Third Edition (WMS-III) (74)
Language	Abbreviated Boston Naming Test (15-BNT) (75)
	Sentence repetition
	Verbal comprehension of commands (simple, semi-complex, complex)
	Phonetic and semantic verbal fluency tasks (76)
Visual gnosis	Poppelreuter-type Overlap Figures (77)
	Lurias Clocks test (78)
	The 15-Object test (79)
Motor praxis	Imitation and ideomotor praxis tasks
	WAIS-III Block Design subtest (73)
Executive functions	Automatic Inhibition subset of the Kurtz Syndrome Test (SKT) (80)
	Similarities and Digit backward subtest of WAIS-III (73)
	Clock Drawing Test (81)
Mood	Hospital Anxiety and Depression Scale (HADS) (82)
Functional abilities	Activities of Daily Living (ADL) (83)
	Instrumental Activities of Daily Living (IADL) (84)
Quality of life	Quality of Life in Alzheimers Disease (QoL-AD) (85)

#### 2.4.3 Nursing evaluation

The evaluation will be performed at the nursing station at Ace and includes the collection of anthropometric data such as weight, height, abdominal circumference, and blood pressure, providing key indicators of participants’ physical health. Blood samples are collected for genetic analyses at baseline and for metabolic and proteomic studies at both baseline and follow-up assessments. Additionally, the study incorporates validated tools to assess health risks and comorbidities. Together, these measures ensure a comprehensive evaluation of participants’ biological and physical health profiles, supporting the exploration of potential associations between these factors and cognitive decline.

#### 2.4.4 MRI acquisition and preprocessing

A Siemens MAGNETOM VIDA 3T scanner (Erlangen, Germany) equipped with a 32-channel head coil will be utilised at Clínica Corachan facilities in Barcelona. Anatomical images for volumetric analyses will be acquired using a T1-weighted 3D MPRAGE sequence, providing high-resolution structural data. Diffusion tensor imaging will be performed using an EPI sequence with 64 diffusion directions to assess white matter integrity. Additional sequences will be employed to detect ischemic damage and microbleeds, enabling a comprehensive evaluation of vascular contributions to cognitive impairment. All acquired images will be processed and analysed at the Neuroimaging Lab at Ace, ensuring consistency and quality in image interpretation and data extraction.

#### 2.4.5 Fluid biomarkers acquisition and measurements

Several biospecimen samples are collected on the same day, forming part of the biobank at Ace, which is officially registered with Instituto de Salud Carlos III (code C.0000299). Ace's samples collection protocols have been approved by the Clinical Research Ethics Commission of the Hospital Clinic (Barcelona, Spain, reference num: HCB/2014/0494) in accordance with the Declaration of Helsinki and the current Spanish regulations in the field of biomedical research (law 14/2007, royal decree 1716/2011).

Blood samples are collected in polypropylene tubes with EDTA (BD Vacutainer). Plasma is separated by centrifugation, aliquoted, and stored at −80°C until use. CSF is obtained through lumbar puncture, performed by an experienced neurologist at Ace following established consensus recommendations. The procedure is carried out on fasting patients, who are seated and locally anesthetised with 1% subcutaneous mepivacaine. A total of 13 mL of CSF is collected using polypropylene tubes (Sarstedt Ref 62.610.018). CSF samples are centrifuged (2,000× g, 10 min, 4°C). The resulting supernatant is divided into aliquots and stored at −80°C for subsequent analyses. This collection protocol strictly adheres to the recommendations outlined by the Alzheimer's Biomarker Standardization Initiative (40), ensuring high-quality and standardised sample handling for biomarker evaluation. AT(N) classification will utilise core CSF biomarkers, including amyloid-β40 (Aβ40), amyloid-β42 (Aβ42), phosphorylated tau (pTau181), and total tau (tTau). Positivity thresholds follow the cut-offs established by the CSF program at Ace Alzheimer Center Barcelona (41), these cut-off values are based on validated population-derived distributions and have been previously applied in clinical and research cohorts to characterize neurodegenerative progression. In addition, plasma biomarkers (pTau217) are measured for research purposes (42).

#### 2.4.6 Genotyping

Genomic DNA is extracted from buffy coat samples using the Maxwell RSC 48 instrument (Promega) with the AS1540 DNA extraction kit, ensuring high-quality samples for genetic analysis, and targeting genotype variants included in the latest Polygenic Risk Score (PRS) for AD, including *APOE*. *APOE* genotypes are determined using fluorogenic allele-specific oligonucleotide probes via the TaqMan assay (Life Technologies, Spain) for the *APOE* genetic variants rs7412 (Test ID: C_904973_10) and rs429358 (Test ID: C_3084793_20). For the TaqMan assays, polymerase chain reaction (PCR) and real-time fluorescence measurements are performed on the QuantStudio3 real-time PCR system (Thermo Fisher Scientific, Spain) utilizing the TaqMan Universal Master Mix system (Ref: 4364341, Life Technologies, Spain), strictly following the manufacturer's protocol. The PCR protocol consists of an initial read step at 60°C for 30 s, followed by 40 cycles comprising an initial denaturation at 95°C for 10 min, a denaturation step at 95°C for 15 s, and annealing at 60°C for 1 min. The process concludes with a final post-read step at 60°C for 30 s. Genotypes are determined through the Genotyping application available on Thermo Fisher Cloud, employing cluster analysis to ensure accurate identification. This standardised methodology provides reliable *APOE* genotyping for downstream analyses. Additionally, DNA from the same participants is sent for genotyping using the Axiom 815K Spanish biobank array (according to the manufacturer's instructions—Axiom™ 2.0 Assay Manual Workflow, Thermo Fisher) at the Spanish National Center for Genotyping (CeGEN, Santiago de Compostela, Spain). Details on genotyping and quality control procedures are provided elsewhere (43). PRS were calculated following (44), using a list of 83 single nucleotide polymorphisms.

#### 2.4.7 Spontaneous speech

The evaluation of spontaneous speech is an integral part of the assessment protocol provided by Punto Health (45). Ace has extensive experience in administering and processing of spontaneous speech protocols, as demonstrated in recent publications from our group (23, 24).

This assessment is designed to be conducted exclusively in a remote setting, such as the participant's home, ensuring convenience and minimizing disruptions to daily life. The protocol is administered via a mobile application that is downloaded onto the participant's or caregiver's device. The speech assessment involves a picture description task where they describe a given image for one minute, followed by a semantic category fluency task, listing as many items as possible within a given category, such as fruits or professions, within a minute. Finally, open-ended questions prompt participants to describe personal experiences and daily routines. All three tasks are completed within 3 minutes.

### 2.5 Standardised evaluation protocol

A common evaluation protocol has been developed within the project to ensure methodological consistency and enable meaningful cross-site comparisons (see Table 3 for details on self-administered questionnaires). This standardised approach allows for the integration of data from diverse pilot sites while preserving inter-comparability and scientific rigor.

**Table 3:** Self-administered questionnaires of COMFORTage-Ace (basal, one-year and two-year follow-up).

Category	Measurement Tool/Description
Quality of life	QoL-AD (85)
Depression	HADS (82)
	Geriatric Depression Scale (86)
Sleep quality	Pittsburgh Sleep Quality Index (PSQI) (87)
Satisfaction with intervention	Questionnaire for User Interaction Satisfaction (QUIS) (88)
Alcohol consumption	Alcohol Use Disorders Identification Test (AUDIT) (89)
Social isolation	UCLA Loneliness Scale (90)
IADL	Lawton IADL scale (84)
Daily living activities	ADL (83)
Physical activity	International physical activity questionnaire (IPAQ) (91)
Nutritional assessment	Mini Nutritional Assessment (MNA) (92)

QoL-AD, quality of life in alzheimer's disease; HADS, hospital anxiety and depression scale; IADL, instrumental activities of daily living; ADL, activities of daily living.

The collection of genetic data, CSF and plasma samples, as well as MRI scans, is carried out in strict adherence to bioethics committee–approved protocols and in compliance with relevant national and international regulations, including the GDPR. Blood samples are obtained by licenced nurses in a designated clinical area, and CSF procedures are performed by a neurologist or anesthesiologist at Ace. Prior to any procedure, participants receive detailed information and are given sufficient time to ask questions, ensuring informed understanding and comfort.

Given the cognitive challenges faced by many participants, the completion of questionnaires is designed to be flexible: participants are allowed several days and may receive support from trained staff as needed. To minimize any perception of coercion and uphold ethical standards, the Participant Information Sheet explicitly states that participation is voluntary and that individuals may withdraw at any time without consequences to their clinical care. Furthermore, it is made clear that the clinical procedures performed in the context of the project are independent of the study's research objectives. This ensures full respect for participant autonomy and safeguards their rights throughout the research process.

### 2.6 Intervention plan

The active group (*n* = 50) will participate in in-person visits, using the cognitive and functional stimulation tools developed by the Consortium and completing follow-up assessments when needed. Sessions will be conducted by a neuropsychologist and delivered twice per week in one-hour format, ensuring sufficient intensity and structure to promote cognitive engagement. Participants will attend these sessions in small groups of approximately five to seven participants, allowing the neuropsychologist to combine individualised attention with the therapeutic benefits of group interaction. The content for each participant will be customised by combining recommendations from the VHP and input from the neuropsychologist conducting the sessions, who will direct the session intervention. The sessions will take place at Ace's facilities, ensuring a controlled and supportive environment tailored to participants’ needs. This structured schedule provides a consistent therapeutic framework during the first year of follow-up. Importantly, needed medication regimens will be maintained throughout the intervention, ensuring no disruption of clinical care.

The protocol will integrate individualised and group-based approaches, with activities tailored to each participant's functional abilities and progress. For participants with lower digital literacy, face-to-face support will play a pivotal role, ensuring comprehension and fostering a confident and engaging experience. Participants demonstrating sufficient motivation and engagement may have the option to continue activities at home, supported by direct and remote monitoring from assigned professionals. This ensures consistent guidance and task adaptation based on evolving needs. The digital tools will provide multiple outputs (*i.e.*, dosage, time of exposure, performance metrics) which will be analysed during and after the intervention. These outputs will serve a dual purpose: enhancing the dynamic functionality of the VHP and providing critical data to evaluate the intervention's efficacy within this study.

In the Pilot 3 study, the tools used to achieve the functional stimulation targets were carefully selected by the researchers at Ace. They evaluated different options provided by the consortium's technology partners to identify those best suited to the specific needs and characteristics of their participants with cognitive decline. Designed for use by both participants and professionals, these tools facilitate seamless data integration and effectively support the program's goals. The tools used in this pilot study include:

Healthentia (46). Healthentia is a class IIa medical device, intended for supporting subject telemonitoring, decision-making and virtual coaching (47, 48). The collection of physiological data includes heart rate, blood pressure, oxygen saturation, and weight, directly transmitted to care providers via automated electronic means in combination with validated internet of things devices (when available for other clinical partners, not for our pilot). The information collected is further enriched with the transmission of patient's outcomes and outcome scores related to patient's health status, health-affecting factors, health-related quality of life, disease knowledge and adherence to treatment, through validated questionnaires. Participants capable of remotely interacting with the tool beyond face-to-face sessions tend to report more frequently, contributing regular (daily or weekly) data that can inform timely care plan adjustments. All these parameters are visualised (subjects-based dashboards) and mathematically treated (trends analysis, alerts) for supporting experts’ decision-making procedures.
Eligence (49). Eligence is an online platform designed for brain training through interactive games, created by specialists in neuropsychology aimed at individuals in the early stages of dementia, for rehabilitation and prevention of cognitive decline. It features 40+ games and activities that exercise the basic cognitive skills such as memory, reasoning, attention, orientation, executive functions, construction skills, language. Through Eligence, healthcare professionals can create personalised training sessions for individuals and groups, monitor performance in real-time and easily generate detailed evaluation reports. It is accessible from anywhere and any device with an internet connection.
Linguistic Games (50). A novel cognitive and linguistic tool has been designed upon the main objective to exploit multiple cognitive processes that are involved in language performance and speech production and comprehension. The initial functionality of the mock-up tool was in 2D, but also some subtasks have been designed in 3D to introduce higher cognitive-processing demands; however, the tool is not intended as different versions for separate groups but rather as graded levels of difficulty applicable across healthy adults, adults with subjective cognitive decline, and those with mild cognitive impairment. These novel linguistic tests can assess different cognitive abilities through the vehicle of language competence such as attention, memory, language, psychomotor abilities, processing speed and executive functions. The linguistic mock-up has been designed based on Usability and Accessibility Guidelines (51–55), while realistic contexts from older adults’ daily routines have been incorporated for example tasks based on vocabulary or short scenarios related to household chores, daily mobility, travel planning or participation in social events (56).

The data generated by these three digital tools, as well as the clinical, neuropsychological, or biological data relevant to the project—whether retrospective (historical data from Ace) or prospective (related to the follow-up of the two study groups)—will be shared with the Consortium using the VHP. These multimodal data will feed into the platform and serve as input for AI algorithms and PDTs, which aim to predict individual health trajectories and propose tailored interventions. This process exemplifies the integration of digital tools, clinical knowledge, and computational models into a unified, adaptive care system. Audio recordings from the spontaneous speech protocol will not be shared for confidentiality reasons.

The active group will complete the cognitive and functional stimulation intervention at the one-year follow-up. In parallel, the control group will follow the same assessment protocol as the active group, ensuring consistency and comparability in the assessment process. However, participants in the control group will not participate in the stimulation activities provided through the digital platforms developed by the project.

### 2.7 Analysis plan

The primary objective of Pilot 3 is to compare cognitive decline between the active and control groups after a two-year follow-up period. The neuropsychological structure underlying the primary outcome has been previously defined and validated by an expert panel of neuropsychologists (26, 27). Based on this structure, five composite scores will be derived using Structural Equation Modelling, following the methodology described in (23). To evaluate the impact of the intervention across two conditions on these neuropsychological composites, mixed models will be employed. These models will include three time points (baseline, one-year follow-up and two-year follow-up) and will also be applied to secondary outcome measures, such as quality of life, physical activity, and satisfaction with the intervention. Alterations in plasma biomarkers, including pTau181 and pTau217, will adhere to the same analytical framework, allowing for a comprehensive evaluation of both cognitive and biological markers.

In addition, the study will explore modulating effects, such as biological factors (AD-related biomarkers in CSF, PRS, *APOE*, MRI parameters, plasma biomarkers, comorbidities, and medication use) through interaction analyses within the mixed models. Associations between spontaneous speech measures, neuropsychological composites, and plasma biomarkers will be examined using regression models and advanced machine learning techniques. This aspect of the study aims to identify the most predictive parameters of pre- and post-intervention slopes across the five neuropsychological composites. By leveraging these predictive models, the study seeks to advance the understanding of how specific variables contribute to cognitive decline and its modulation, ultimately informing precision medicine approaches for dementia care.

Building upon this analytical framework, a comprehensive ML approach will be integrated to enhance predictive modelling and improve the understanding of cognitive decline in response to intervention. In contrast to earlier work that relied primarily on shallow or ensemble learning methods applied to acoustic features, the present study will evaluate a broader range of modelling strategies. Acoustic, prosodic, and temporal speech features will be analysed alongside linguistic features derived from automatic transcription, including lexical, semantic, syntactic, fluency-related, and sentiment-related measures (57). Deep-learning architectures—such as convolutional neural networks and autoencoder-based models—will be explored for both acoustic representations and linguistic embeddings. The potential use of large language models (LLMs) will also be examined to extract contextual and semantic information from transcripts (58).

To manage the complexity of high-dimensional data, feature engineering and dimensionality reduction techniques will be applied. Specifically, wrapper-based feature selection methods and autoencoders will be tested to extract the most relevant predictors while minimizing redundancy and noise. A regression-based predictive framework will be used to evaluate the contribution of cognitive, biological, and speech-derived parameters, incorporating ensemble methods such as Random Forests and Gradient Boosting.

To improve model interpretability, explainable AI techniques, such as SHapley Additive exPlanations (SHAP) (59), will be employed to identify the key factors driving cognitive outcomes. Furthermore, longitudinal analyses will be conducted to track cognitive changes over time, enabling an assessment of individual responses to interventions and facilitating the early identification of cognitive decline. This machine learning-driven approach is expected to complement traditional statistical methods, enhancing both the accuracy and interpretability of predictive models in the context of dementia research.

In addition to these methodological goals, the study will also assess how the integration of digital health tools within the VHP supports clinicians in decision-making processes. The increased frequency, granularity, and clarity of data provided by these tools are expected to improve clinical insight, facilitate earlier detection of cognitive decline, and enable more timely and personalised interventions, thereby reinforcing trust in AI-assisted models as complementary assets in routine care.

## 3 Discussion

The COMFORTage project exemplifies a transformative and multidisciplinary effort to advance dementia care through the integration of cutting-edge digital technologies, biomarker-based research, and precision medicine strategies. By bringing together a consortium of 39 organizations across 12 countries, this initiative utilises a wide range of expertise and resources, ensuring scalability and external validity of its findings across various healthcare systems.

One of the project's distinguishing features lies in its advanced technological infrastructure. The VHP integrates multidimensional health data, encompassing all key aspects of patient information, supported by the incorporation of PDTs to enhance precision and personalization (60). These strategies enable adaptive decision-making, optimize interventions in real-time, and provide a secure, scalable framework for patient-centred care. Features such as explainable AI, gamified interventions, and educational components further improve engagement and usability (61, 62).

Although the COMFORTage project addresses a wide range of age-related conditions and populations, Pilot 3, led by Ace, focuses specifically on individuals with MCI and mild AD dementia. This pilot assesses the effectiveness of tailored cognitive and functional stimulation interventions, aiming to enhance patient outcomes and inform scalable care strategies. Through a randomised, open-label trial design, participants are divided into active intervention and control groups to rigorously assess outcomes.

By combining retrospective datasets—including thousands of clinical, neuropsychological, genetic, and imaging records—with prospective longitudinal assessments, Pilot 3 will generate a rich dataset for predictive modelling. These assessments incorporate both traditional biomarkers, such as plasma and MRI, and non-invasive biomarkers, including spontaneous speech analysis, which detects subtle cognitive changes through linguistic parameters such as lexical fluency and syntactic complexity (23, 63). Additionally, genetic variables such as PRS and the presence of the *APOE ε*4 allele are included, further enriching the dataset and enhancing its predictive capabilities (43).

Findings for Pilot 3 are contextualised within the broader COMFORTage framework, which encompasses complementary pilots addressing other aspects of age-related health challenges. These include systemic approaches to care delivery, physical health and mobility, and prevention strategies targeting broader ageing populations. Together, these efforts refine precision medicine approaches, ensuring interventions are not only effective but also scalable and adaptable to various healthcare systems and patient demographics (64, 65). Although the Ace dataset will include 100 participants, its contribution gains substantial relevance when combined with the broader multimodal datasets generated across the COMFORTage consortium. These larger and more diverse cohorts provide a powerful framework for validating AI-driven models and for situating the Ace data within a wider European context. Importantly, the consortium-wide datasets complement established public cohorts—such as ADNI and OASIS (66, 67)—by contributing real-world clinical information, fluid biomarkers, spontaneous speech recordings, and digital-platform activity collected within community-based intervention settings.

Studies suggest that multimodal stimulation programs combining cognitive, social, and digital activities may stabilise or modestly enhance cognitive trajectories in individuals with MCI and early AD, particularly in executive and attentional domains (68, 69). Likewise, plasma biomarkers show strong associations with longitudinal cognitive decline and may be expected to correlate with neuropsychological slopes (70). MRI-based measures have also demonstrated additive predictive value when integrated with neuropsychological data (71). Given these trends, we anticipate that the multimodal dataset generated in Pilot 3 will support more accurate, personalised prediction models within the VHP.

Despite its innovative and comprehensive design, the COMFORTage project faces several key challenges that may influence its overall impact and scalability (72). Among these, the digital divide remains a significant concern, particularly in terms of unequal access to technology and varying levels of digital literacy among older adults. To mitigate this risk, structured onboarding sessions and guided pre-training may be used to help participants become familiar with digital platforms, reducing early practice effects and enhancing long-term engagement.

Likewise, regional differences in healthcare infrastructure and resource availability may affect the feasibility of implementing standardised digital platforms at scale. Another important consideration involves ethical and legal aspects related to the management of sensitive data, especially when dealing with genetic information and biomarker profiles. Ensuring data privacy, patient consent, and compliance with regulatory frameworks like GDPR is crucial to maintain public trust and protect participants’ rights.

From a methodological perspective, Pilot 3 incorporates several overlapping digital interventions—such as Healthentia, Eligence, and the Linguistic Games tools—that deliver cognitive stimulation, functional training, and patient monitoring. Although this multimodal approach enhances the realism and relevance of the intervention, it also poses analytical challenges: it is difficult to disentangle the specific contribution of each platform to the observed outcomes. Improvements in cognitive function or quality of life may stem from the synergistic effects of multiple factors, including physical activity, digital engagement, social interaction, and cognitive challenge.

This limitation is intrinsic to many complex, integrative interventions and reflects the multifactorial nature of neurodegenerative conditions. While the study does not allow for precise attribution of effects to single components, it aligns with the project's overarching goal: to promote holistic, patient-centred care through scalable, real-world strategies. Importantly, the availability of extensive clinical, demographic, and biological data enables us to account for alternative explanations and rule out several potential confounders when interpreting the observed effects. Future research may benefit from experimental designs such as factorial or dismantling trials to further isolate and quantify the contributions of individual intervention elements within broader care models.

In conclusion, while COMFORTage addresses a diverse array of age-related health challenges, Pilot 3's emphasis on cognitive and functional stimulation interventions, supported by advanced biomarker analyses, speech-derived metrics, and digital health tools, is expected to make a significant contribution to the project's overarching goals. By integrating multimodal data within a precision-medicine framework, this pilot is well positioned to inform scalable, evidence-based strategies for early intervention in cognitive decline. Collectively, the interconnected pilots offer a cohesive foundation for future innovation in dementia care, supporting real-world implementation across diverse populations and healthcare settings.

## COMFORTage consortium members

Please see the Supplementary Material for consortium members.
